# Linear Atrophoderma of Moulin: A Case Report

**DOI:** 10.31729/jnma.8648

**Published:** 2024-07-31

**Authors:** Anu Duwal, Sunil Timilsina, Sudarshan Pokhrel

**Affiliations:** 1 Department of Dermatology and Venereology, Nepalese Army Institute of Health Sciences, Kathmandu, Nepal; 2 Department of Emergency Medicine, Dirghiayu Guru Hospital and Research Center, Kathmandu, Nepal; 3 Department of Family Medicine, Nepal Armed Police Force Hospital, Kathmandu, Nepal

**Keywords:** *artophoderma*, *line of blaschko*, *unilateral rash*

## Abstract

Linear atrophoderma of Moulin is a rare skin condition that is characterized by the development of one or more atrophic patches or depressions in the skin. These patches are usually located on the trunk, but they can also occur on the arms, legs, and neck. We here present a case of 33-year Nepalese male with brown to black color lesions over the left upper back, abdomen and thigh for the last 7 years. Clinical and dermatopathological findings were similar to the linear atrophoderma of Moulin. To our knowledge, this is the first case of linear atrophoderma of Moulin from Nepal. This case emphasizes the necessity of diagnosing atrophoderma of Moulin and separating it from linear scleroderma due to differences in therapy and prognosis.

## INTRODUCTION

Linear Atrophoderma of Moulin (LAM) is a rare dermatosis with few case reports. It is characterized by the presence of linear, atrophic, and hyperpigmented streaks on the skin.^1^ Although LAM is considered a benign condition, its appearance can cause significant cosmetic concern and psychological distress. The etiology of LAM remains unclear, and its pathogenesis is poorly understood.^2^ It typically presents during adolescence or early adulthood. The streaks usually develop unilaterally on the trunk, back, or extremities, following the lines of Blaschko, which are thought to represent the embryonic migration pathways of the skin.^3^ Till date no effective treatment has been proven. As it is a benign condition, the prognosis is good.^3^ This case report adds to the sparse literature and emphasizes the need for accurate diagnosis and differentiation from similar conditions such as linear scleroderma and Atrophoderma of Pasini.

## CASE REPORT

A 33-year male, without significant medical, surgical, psychosocial and family history presented to the Dermatology Out-patient Department (OPD) with complaints of multiple brown to black-colored patches on the left side of his body. The lesions initially appeared on the left upper back and, over several years, spread to the abdomen, thigh, and arm. These patches were asymptomatic and had remained stable for the past 2 years. Additionally, the patient reported a tingling sensation in both hands for the past 6 years. The tingling was gradual in onset, progressive, and accompanied by slight weakness in the hand. Despite receiving both topical and oral medications six years ago, the symptoms persisted. A skin biopsy was conducted to rule out Hansen's Disease, yielding negative results.

**Figure 1:**
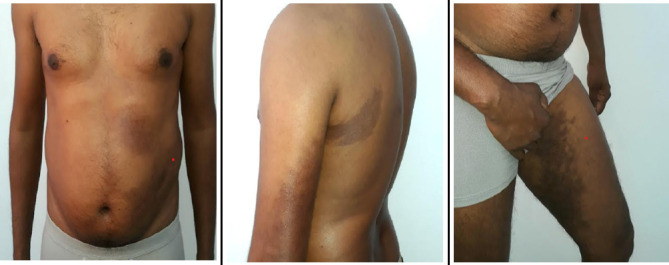
A) Hyperpigmented patches were observed on the left side of the abdomen, and left iliac region, B) Similar lesions on the left upper back and posterolateral aspect of left arm, C) Similar lesions over to the medial aspect of left thigh.

Upon dermatological examination, multiple hyperpigmented macular lesions of varying sizes ranging from 4 x 3 cm to 26 x 5 cm were observed, with slight sclerotic depressions respecting the midline over the left half of the body (Figure 1). Sensory and motor examinations were within normal limits.

**Figure 2:**
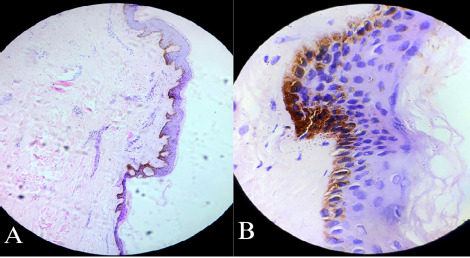
A) Hematoxylin-eosin stain. The epidermis was acanthotic with pigmented basal layer. Dermis had sclerotic collagen with high-up eccrine glands with loss of peri-eccrine fat and sparse perivascular lymphocytic infiltrate, B) Same slide in 100X.

Histopathological analysis of the skin biopsy revealed an acanthotic epidermis with a pigmented basal layer (Figure 2). The dermis exhibited sclerotic collagen, heightened eccrine glands, loss of peri-eccrine fat, and sparse perivascularlymphocyticinfiltrate. Subcutaneous tissue appeared unremarkable.

Fite stain results were negative for AFB (acid-fast bacilli), and the histological impression suggested findings indicative of LAM (Figure 2). Additionally, the Antinuclear Antibody (ANA) was negative.

He was started on a weekly dose of Tab Methotrexate 15 mg, Tab folic acid 5 mg, and Oral Minipulse therapy of Methylprednisolone 32 mg taken on two consecutive days per week for 1 year along with clobetasol propionate once daily and calcipotriol ointment once daily as well. Daily dose steroid was not given in view of its adverse effects on long-term use. He was in regular follow-up for monthly on initial 3 months and once two months thereafter with good compliance of medicine without any adverse effects. At the end of treatment patient had a partial decrease i.e., in size, pigmentation, and atrophy of lesions. Patient lost to follow-up so the post-treatment assessment could not be done.

## DISCUSSION

Linear atrophoderma of Moulin (LAM) was initially documented by Moulin et al in 1992, with only 5 cases observed over a 17-year span. Baumann et al described another case with identical symptoms and proposed the name Linear atrophoderma of Moulin (LAM).^1^ The diagnostic criteria for LAM include Onset at childhood or adolescence, Development of hyperpigmented, slightly atrophic, unilateral lesions following Blaschko lines on the trunk or limbs, Absence of preceding inflammation and subsequent induration or scleroderma, Stable, nonprogressive clinical course without a pattern of remission, Histologic findings showing hyperpigmentation of the basal epidermis and a normal dermis with unaltered connective tissue and elastic fibers.^2^

Opposite to these criteria, Browne-Fisher noted a preceding inflammatory phase, both clinically and histologically, proposing that LAM has an antecedent inflammatory phase that progress hyperpigmentation with atrophy. They suggested two variants: an inflammatory and a non-inflammatory.^3^ Similarly, Utikal et al. reported patients with marked telangiectatic erythema within lesions of linear atrophoderma, suggesting a novel variant.^4^ The exact etiology of LAM remains unknown but is postulated to result from somatic mosaicism during early embryogenesis.^5^ Zahedi et al. hypothesized LAM as an autoimmune pathology after discovering elevated ANA titers and abnormal serum ribonucleoprotein, immunoglobulin M, and anti-SM antibody in a 28-year-old male with LAM.

Histopathologically, LAM is characterized by hyperpigmentation of basal cells, slight thickening of collagen fibers in the dermis, and sparse perivascular lymphocytic infiltrate. Routine histologic examination of LAM specimens typically doesn't show clear signs of dermal atrophy. However, clinically atrophic lesions may result from focal decreases in subcutaneous tissue, as observed in one case.^6^ In our case, dermal findings indicate sclerotic collagen with elevated eccrine glands, supporting the notion that LAM is within the spectrum of morphea.

Conversely, microscopic examination of linear scleroderma reveals thickened and closely packed collagen bundles, alongside atrophic eccrine glands, hair follicles, and peri-appendageal fat deposition, often indicating preceding inflammation. Lesions of Atrophoderma of Pasini and Pierini (APP) present bilaterally with a cliff-drop edge and resemble LAM histologically.^3,5^

Other dermatological conditions following Blaschko lines, such as epidermal naevi, lichen striatus, linear scleroderma, Goltz syndrome, and Blaschkoid dermatitis, exhibit different morphologies.

Currently, there are no proven effective medications for LAM. High-dose penicillin, topical steroids, heparin, and oral potassium benzoate have not demonstrated efficacy.^5^ Zaouak et al. reported successful treatment of a 21-year-old female diagnosed with LAM using methotrexate 20mg/week for 6 months, resulting in significant improvement of skin pigmentation and atrophy which has been similar to our presentation but with a lower dose and longer duration.^7^ There are reports of stabilization of early LAM lesions with partial effectiveness.^2^ Kharkar et al. reported a case treated with intralesional platelet-rich plasmainjectionsshowing partial improvement.^8^ However, topical tacrolimus exhibited no improvement after continuous application for 4 months.^9^

Our patient was satisfied in terms of a decrease in size, hyperpigmentation, and atrophy but since there was only partial improvement after 1 year of use he was not willing to continue it further which was also due to the disease being asymptomatic in nature.

LAM is a rare and under-recognized condition that requires differentiation from other clinical conditions such as linear scleroderma and APP. Tab Methotrexate and Oral mini pulse steroid have shown partial efficacy when used for 1 year duration. Large-scale studies are necessary to establish effective drug therapies for LAM.
